# Evidence of validity and reliability of the adaptive functioning scale for intellectual disability (EFA-DI)

**DOI:** 10.1186/s41155-020-00164-7

**Published:** 2020-10-31

**Authors:** Thais Selau, Mônia Aparecida da Silva, Euclides José de Mendonça Filho, Denise Ruschel Bandeira

**Affiliations:** 1 Graduate Program in Psychology. Universidade Federal do Rio Grande do Sul, Rua Ramiro Barcelos, 2600 sala 120, Bairro Santa Cecilia, Porto Alegre, RS CEP 90035-003 Brazil; 2 Graduate Program in Psychology. Universidade Federal de São João del-Rei, São João del-Rei, MG Brazil; 3 Department of Psychiatry, McGill University, Montreal, Canada

**Keywords:** Adaptive functioning, Intellectual disability, Assessment, Test construction

## Abstract

Intellectual disability (ID) is a developmental disorder characterized by deficits in intellectual functioning and adaptive behavior. The fifth edition of the *Diagnostic and statistical manual of mental disorders* (DSM-5) defines adaptive functioning as a severity measure of ID. The availability of tests in the international context to assess this construct has increased in recent years. In Brazil, however, non-systematic assessment of adaptive functioning, such as through observation and interviews, still predominates. The *Escala de Funcionamento Adaptativo para Deficiência Intelectual EFA-DI* [Adaptive Functioning Scale for Intellectual Disabilities] is a new instrument developed in Brazil to assess the adaptive functioning of 7- to 15-year-old children and support the diagnosis of ID. This study’s objectives were to investigate evidence of validity related to the EFA-DI’s internal structure, criterion validity, and reliability. The psychometric analyses involved two statistical modeling types, confirmatory factor analysis (CFA) and item response theory analysis (IRT). These results highlight the EFA-DI scale’s strong psychometric properties and support its use as a parental report measure of young children’s adaptive functioning. Future studies will be conducted to develop norms of interpretation for the EFA-DI. This study is expected to contribute to the fields of psychological assessment and child development in Brazil.

## Introduction

Intellectual disability (ID) is characterized by deficits in cognitive abilities such as reasoning, problem-solving, planning, abstract thinking, judgment, academic learning, and experiential learning (American Psychiatric Association [APA], 2013). These deficits compromise adaptive functioning: individuals face difficulties adapting independent living and bearing social responsibility in one or more aspects of daily life, including communication, social participation, academic or professional performance, and being independent at home or within the community (AAIDD User's Guide Work Group, 2012; APA, 2013). Deficits are recognized during childhood or adolescence (American Educational Research Association, American Psychological Association, & National Council on Measurement in Education [AERA, APA, & NCME], 2014). Individuals with severe ID are present with delays in motor, linguistic, and social milestones in the early years of life, while limitations among individuals with mild ID may be recognized only later, up to reaching school age (AAIDD, 2012; APA, 2013).

ID is a heterogeneous condition, in which the course of the disease varies, with multiple manifestations and multiple causes, including genetics, organic, social, and environmental causes, possibly in combinations of two or more of these (APA, 2013; World Health Organization [WHO], 2018). It is a stigmatizing condition, causing a significant impact on an individual’s functionality throughout life (AAIDD, 2012; Maulik, Mascarenhas, Mathers, Dua, & Saxena, 2011; Salvador-Carulla et al., 2011). The prevalence of ID is estimated to be 1% of the world population (WHO, 2018).

According to the Diagnostic and Statistical Manual of Mental Disorders (DSM-5), the diagnosis of ID should consider the following criteria: (a) deficits in intellectual functioning, (b) deficits in adaptive functioning, and (c) onset during the developmental period (APA, 2013). Culturally adapted and individually administered intelligence tests with appropriate psychometric validity are available in the literature (AAIDD, 2012; APA, 2013). Scores that are two standard deviations below the population mean indicate a deficit in intellectual functioning, which suggests intellectual disability (APA, 2013).

Intellectual functioning is typically measured with individually administered and psychometrically valid, comprehensive, culturally appropriate, psychometrically sound tests of intelligence. Individuals with intellectual disability have scores of approximately two standard deviations or more below the population mean, including a margin for measurement error (generally +5 points). On tests with a standard deviation of 15 and a mean of 100, this involves a score of 65–75. Clinical training and judgment are required to interpret test results and assess intellectual performance (APA, 2013).

There are currently 31 intelligence tests in Brazil that were favorably assessed by the SATEPSI (Psychological Testing Assessment System; Brazilian Psychology Federal Council, 2018) until the date of this manuscript’s approval. Thus, psychologists may use them to test intellectual functioning. The best known and most widely used tests include the Wechsler scales, which are considered the “gold standard” (Nascimento, Figueiredo, & Araujo, 2018). Among those favorably rated by the SATEPSI are the Wechsler Abbreviated Scale of Intelligence (Wechsler, 1999, adapted by Trentini, Yates, & Heck, 2014) and the Wechsler Intelligence Scale for Children (WISC-IV) (Wechsler, 2004, adapted by Marín Rueda, Angeli dos Santos, & Porto Noronha, 2016).

Along with the measurement of cognitive abilities, the use of individualized valid and culturally adapted instruments is indicated to assess adaptive functioning (AF), in addition to direct observation of behavior and conducting individual interviews (AAIDD, 2012; APA, 2013). These standardized measures of AF can be applied considering multiple informants (e.g., caregivers, family members, teachers) or self-reported, if the severity of the disorder is not an impediment (APA, 2013).

There is a diversity of instruments to assess adaptive functioning in the international context, even though most are not specific for evaluating AF in intellectual disability (Tassé et al., 2012). The instruments most frequently are the Vineland Adaptive Behavior Scales (VABS) (Sparrow, Cicchetti, & Saulnier, 2009) and the Adaptive Behavior Assessment System (ABAS-3) (Harrison & Oakland, 2015). The American Association on Intellectual and Development Disabilities (AAIDD) recently developed the Diagnostic Adaptive Behavior Scale (DABS) to assess adaptive behavior, which was standardized to determine a diagnosis of ID (Tassé et al., 2016). These instruments, however, do not present norms to be used in Brazil. Simultaneously, non-systematic assessment of adaptive functioning seems to predominate (Ferreira & Van Munster, 2015), such as direct behavioral observation and individual interviews, either using multiple informants or self-reported information.

As established by the DSM-5, AF’s assessment is an essential criterion for determining an ID diagnosis (APA, 2013). The severity of the intellectual disability, whether mild, moderate, severe, or profound, is established according to the level of assistance required and the level of impairment in adaptive functioning (APA, 2013). In addition to the diagnosis, it is essential to assess AF domains that are most affected or retained among individuals with ID to plan interventions, monitor their clinical progression, and determine the type of assistance that will be required for a social inclusion process (AAIDD, 2012; Tassé et al., 2012). This knowledge is also necessary to determine the level of assistance patients require (AAIDD, 2012; APA, 2013).

Early and continuing interventions can improve individuals’ quality of life with ID (APA, 2013; Tassé et al., 2012). The level of support provided to older children and adults can enable these individuals to fully participate in daily tasks and improve their adaptive functioning (APA, 2013). Adaptive behavior can improve due to the acquisition of new skills or contingent support and uninterrupted interventions (APA, 2013). Thus, it is necessary to investigate AF within an ID context considering the scarcity of valid instruments for the Brazilian population. In this sense, there is a clear need to invest in studies to develop and validate AF instruments.

The *Escala de Funcionamento Adaptativo para Deficiência Intelectual EFA-DI* (Adaptive Functioning Scale for Intellectual Disability) was designed by Selau, Silva, and Bandeira (2020) to assess the adaptive functioning of 7- to 15-year-old children. This study presents the procedures used to investigate EFA-DI’s psychometric evidence. More specifically, validity concerning the scale’s internal structure and external variables and its reliability were investigated. The study’s objective was to accumulate evidence regarding validity as recommended by the American Educational Research Association, American Psychological Association, & National Council on Measurement in Education [AERA, APA, & NCME], (2014)

## Method

### Participants

A total of 565 primary caregivers (fathers, mothers, grandmothers, grandfathers, aunts, and uncles) of children and adolescents within the age group covered by the EFA-DI (7 to 15 years old) participated in the study. Sixteen cases were excluded because they did not complete at least 50% of the EFA-DI. Hence, the final sample was composed of 549 respondents. Fifty-four percent of them were caregivers of male children/adolescents aged 11.15 years old on average (SD = 2.59).

The convenience sample was composed of children/adolescents’ primary caregivers with intellectual disability (clinical sample) and parents of children/adolescents with typical development (non-clinical sample). A total of 382 (83.2%) participants did not report an ID diagnosis (typical development group—non-clinical), while 163 cases reported different levels of ID (clinical group). The clinical group was 11.32 years old (SD = 1.41), while the non-clinical group was 10.73 years old (SD = 2.68) on average, with no significant difference. Other potential comorbidities, such as autism spectrum disorder (ASD), Down syndrome, cerebral palsy, and motor coordination development disorder, were investigated. Table 1 presents the distribution of the sample data according to the diagnoses reported.

**Table 1:** Distribution according to the diagnoses reported by the clinical sample respondents

	*F*	%
Diagnosis		
Autism spectrum disorder (ASD)	67	40.1
Down syndrome	9	5.4
Cerebral palsy	42	25.1
Developmental coordination disorder	23	13.8
Mild intellectual disability	89	53.3
Moderate intellectual disability	44	26.3
Severe or profound intellectual disability	21	12.6
Unspecified intellectual disability	9	5.4

Regarding developmental characteristics, only 24% had complications such as cardiorespiratory problems at birth, 61.2% were born by cesarean, and 53.8% were born with 38 to 40 weeks of pregnancy.

The ages of the respondents, that is, the caregivers of the children and adolescents, ranged from 18 to 80 years old, with an average age of 41.7 years old (SD = 10.2). The average number of children per family was 1.9 (SD = 1.0), with a maximum of seven children. Most respondents were mothers (78.2%) of the children and adolescents and lived in Rio Grande do Sul (78.4%) and Minas Gerais (17.23%), Brazil. Most participants (43.1%) had completed high school regarding the educational level, and most had a paid job (50.5%). Most respondents reported a family income of up to two times the minimum wage (37.2%).

### Instruments

Adaptive Functioning Scale for Intellectual Disability (*Escala de Funcionamento Adaptativo para Deficiência Intelectual*, EFA-DI, in the original) assesses the adaptive functioning of 7- to 15-year-old children. The conceptual domain comprises 12 items, the social domain is composed of 16 items, and the practical domain consists of 24 items. The conceptual domain addresses competency in terms of academic knowledge and problem-solving skills. Examples of items in the conceptual domain involve can understand the rules of a game and play correctly (C9) and knows how to read like children/teenagers his age (that is, has similar reading skills) (C2). The social domain addresses social intelligence, that is, how competent individuals are in terms of social relationships and others’ perceptions. Examples include can perceive people’s bad intentions (S14) and understands expressions with a different meaning than it seems (e.g., I kept you in my heart) (S3). The practical domain assesses learning capacity and self-management in various contexts of life. Examples include put on and take off your clothes (includes clothes with buttons and zippers or zippers) (P15) and serve your food during meals (that is, take the food out of the pan with a spoon and put it on the plate) (P7).

Answers are provided on a three-point Likert scale: 1—yes; 2—only with assistance; 3—no. The respondent may also choose “I do not know” if s/he is unable to select one of the three options available.

The scale’s development involved five stages: the theoretical foundation, the establishment of the dimensions and items of the preliminary version, the analysis of items by four expert judges, the semantic analysis of items by the target population, and a pilot study (Selau, Silva, and Bandeira, 2020). The instrument has 52 items, divided into conceptual, social, and practical domains according to the theoretical conceptualization of adaptive functioning adopted by the Diagnostic and Statistical Manual of Mental Disorders (DSM-5).

Sociodemographic and clinical characteristic questionnaire is composed of questions addressing sociodemographic and clinical characteristics of children and their respective caregivers, which, according to the literature, influence child development. The variables include socioeconomic status, age, sex, birth order, and number of siblings, among others. Additionally, information regarding other factors reported in the literature as having the potential to influence intellectual development was also collected.

### Data collection procedures

Cross-sectional data were collected from a convenience sample using a quantitative approach. The clinical sample was recruited from private and public health facilities, while the control sample was composed of the parents of students attending public and private schools.

Data were collected in person in the metropolitan region of Porto Alegre, RS (70.87% of data), and in São João del-Rei, MG (14.75%), Brazil. Data were also collected online using the Survey Monkey platform (14.38%). The research was disseminated on the internet, and the sample was by convenience.

The participants were asked to complete the EFA-DI and the sociodemographic and clinical characteristic questionnaire. Data were collected at the facilities’ rooms or the participants’ homes. In the latter case, the participants took the scale and questionnaire to complete them at home and later returned them to the study’s team. When the researcher was present while the participants answered the EFA, additional care was taken to only answer specific doubts without interfering or attributing a score to the participants’ items.

### Ethical procedures

The Institutional Review Board at the Institute of Psychology at the Federal University of Rio Grande do Sul approved the project (protocol number 2,468,130). All the participants were ensured of their data confidentiality and informed they could withdraw from the study at any time. The participants received clarification regarding the study’s objectives and procedures and signed free and informed consent forms.

### Data analysis

Data were analyzed with SPSS to statistical programs, and the participants who did not complete at least 50% of the EFA-DI were excluded. “Yes” answers scored two points, “Only with assistance” scored one point, and “No” answers scored zero; thus, the higher the score, the greater the AF. Some respondents did not understand the option “Not applicable—NA”. These individuals chose this option when the child/adolescent did not present a given behavior instead of choosing “No”. For this reason, NA answers were considered missing data for analysis and subsequently were permanently removed from the scale. Descriptive statistics were used to analyze the sample’s characteristics, data concerning children’s development, and the respondents’ characteristics.

The psychometric analyses involved two statistical modeling types, confirmatory factor analysis (CFA; Brown, 2015) and item response theory analysis (IRT; de Ayala, 2009). The unidimensionality of each of the EFA-DI domains was verified separately using CFA. A model was then specified in which the scale domains would provide an overall score of adaptive functioning through a second-order latent variable. Hence, the matrix of the polychoric correlation of the data originating from each of the scale domains was submitted to the weighted least squares estimation method. This method was chosen because normality is not assumed and because it offers more accurate and less biased estimates for categorical indicators of an ordinal level (Flora & Curran, 2004). The model’s goodness of fit was assessed using the CFI (comparative fit index), TLI (Tucker-Lewis index), RMSEA (root mean square error of approximation), and SRMR (standardized root mean square residual). RMSEA and SRMR parameters below 0.05 indicate a good fit, while parameters below 0.08 indicate an acceptable fit. CFI and TLI above 0.95 suggest excellent fit, while above 0.90 indicates a satisfactory quality of fit (Hu & Bentler, 1999).

The investigation concerning items’ adequacy in the measurement model was performed using item response theory (IRT) analysis. The quality of items was investigated via analysis of residuals using the infit mean-square indicator. The infit mean-square assesses the discrepancy between the fitted values from the measurement model and the weighted observed responses. This index weighs more heavily on the performance close to the item’s difficulty level, which results in lower sensitivity to residuals in extreme situations or situations more distant from the item’s difficulty (Linacre, 2002; Linacre & Wright, 1994). According to Linacre (2002), items with infit mean-square close to 1 are the ones that contribute most to a measure’s development. Values below 0.5 or between 1.5 and 2 are less productive but do not degrade the measure’s quality. Values above 2, however, represent noise or variance not explained by the factor effect. Therefore, as a criterion to suggest items appropriate for the EFA-DI’s domains, infit mean-square values considered acceptable were values between 0.5 and 1.5. The outfit mean-square was not considered an indicator that could determine the quality of items because it is a measure that is more sensitive to unexpected answers and because it represents a lower impact on the measurement system (Linacre, 2002).

The adequacy of the set of items to the measurement model was also assessed using the item-person map. The map illustrates the disposition of people’s continuum of skills regarding the continuum of items’ difficulty. Hence, it enables inferring what part of the latent trait the parameters are more accurate (Bond & Fox, 2015). The graphic representation allows verification of whether the scale presents a ceiling effect, that is, whether there are many people with higher skills who are not discriminated by the scale’s items, or a floor effect, when there is a lack of easy items to discriminate individuals with lower skills (Wang, Byers, & Velozo, 2008).

Additionally, Cronbach’s alpha and McDonald’s omega (Dunn, Baguley, & Brunsden, 2014) were used to assess the internal consistency of each of the EFA-DI domains (Olsson, 1979). Considering that the items were completed on a three-point Likert scale, the internal consistency indexes were calculated based on the items’ polychoric correlations (Olsson, 1979). The coefficients were considered adequate if above 0.7, as recommended by the [APA] (2014).

For the analysis of the EFA-DI’s criterion validity, differences between subsamples of the non-clinical, mild, moderate, and severe/profound intellectual disability groups were investigated using analysis of covariance (ANCOVA) adjusting for participants’ sex and age. Due to the violation of parametric assumptions (asymmetry of EFA-DI scores), confidence intervals and statistical inference were estimated using robust ANCOVA (Field, Miles, & Field, 2012) with 1000 bootstrapped random samples. Adjustment for multiple comparisons was performed with Bonferroni correction.

Data were analyzed using several statistical software packages. For confirmatory factor analysis, the *lavaan* package (Rosseel, 2012) from the R statistical environment (R Core Team, 2019) was used. Winsteps (V3.7; Linacre, 2010) was used to estimate EFA-DI IRT parameters, and robust ANCOVAs with bootstrap resampling were performed to investigate mean differences between typical and diagnosis groups using the Statistical Package for Social Sciences (SPSS; V18).

## Results

As expected, the descriptive analysis of items indicated a distribution of answers with strong negative asymmetry. This pattern was expected since 83.2% of the sample comprises individuals with typical development; hence, a high prevalence of “Yes” answers—the child/adolescent performs a given task without difficulty or assistance—was observed.

CFA indicated the unidimensionality of the model and of each EFA-DI’s domains, considering an overall factor of second-order adaptive functioning with satisfactory fit indexes. Internal consistency analysis showed domains with high reliability. Cronbach’s alpha ranged from 0.93 in the social domain to 0.98 in the overall domain of adaptive functioning. McDonald’s omega composite reliability also presented optimal values, ranging from 0.94 to 0.99 (Table 2).

**Table 2:** Confirmatory factor analysis fit indexes and internal consistency of EFA-DI’s domains and general scale

	CFA fit indexes					Internal consistency	
Models	χ^2^ (*gl*)	CFI	TLI	SRMR	RMSEA	Alpha	Omega
Social	290.2* (88)	0.97	0.97	0.06	0.07	0.93	0.94
Conceptual	159.7* (54)	0.99	0.99	0.02	0.06	0.97	0.98
Practical	545.2* (250)	0.99	0.99	0.03	0.05	0.97	0.97
Second-order factor of general adaptive functioning	1850.6* (1219)	0.99	0.99	0.04	0.03	0.98	0.99

*CFI* Comparative fit index (acceptable value > 0.90), *TLI* Tucker-Lewis index (acceptable value > 0.90), *RMSEA* Root mean square error of approximation (acceptable value < 0.08), *SRMR* Standardized root mean square residual (acceptable value < 0.08)

*p < 0.05

The IRT analysis indicated that item S4—*uses gestures to communicate his/her needs and desires* (*e.g.*, *wags a finger to say no*; *points to something s/he wants*) and item C12—*remains attentive in routine tasks* (*that is, does not lose focus during tasks*) —did not fit well to the measurement model. Item S4 presented an infit value equal to 2.72, and item C12, an infit value equal to 2.07 and lower factor loading of the conceptual domain’s items (0.77, *p* < 0.01). Thus, both items were considered problematic and were excluded. A new round of analyses was performed, indicating that after exclusions, the fit indexes of the confirmatory analyses remained adequate (Table 2).

All the EFA-DI final version items presented high factor loadings with their factors (Table 3). They ranged from 0.75 to 0.91 (*M* = 0.83) for the social domains, 0.85 to 0.97 (*M* = 0.91) for the practical domain, and 0.91 to 0.99 (*M* = 0.95) for the conceptual domain. Regarding the model considering the overall adaptive functioning factor, the factor loadings ranged from 0.76 to 0.99 (*M* = 0.89), see Table 3.

**Table 3:** Parameters of confirmatory factor analysis and item response item analysis

Item	Domains						Second-order factor of general adaptive functioning	
	Social		Practical		Conceptual			
	Factor loading	Infit	Factor loading	Infit	Factor loading	Infit	Factor loading	Infit
S1	0.85	0.98					0.82	1.34
S2	0.82	1.09					0.82	1.45
S3	0.80	1.09					0.79	1.49
S5	0.87	0.88					0.90	1.10
S6	0.81	1.16					0.82	1.48
S7	0.89	0.95					0.96	0.94
S8	0.72	1.34					0.76	1.44
S9	0.88	0.91					0.88	1.40
S10	0.83	0.87					0.82	1.01
S11	0.92	0.69					0.91	0.93
S12	0.91	0.73					0.95	0.97
S13	0.82	1.00					0.84	1.36
S14	0.76	1.23					0.78	1.47
S15	0.86	0.97					0.88	1.32
S16	0.75	1.23					0.76	1.50
C1					0.94	1.06	0.94	0.7
C2					0.99	0.79	0.99	0.78
C3					0.98	0.91	0.99	0.8
C4					0.93	1.35	0.94	0.95
C5					0.98	0.57	0.98	0.64
C6					0.95	1.01	0.95	0.89
C7					0.91	1.50	0.93	1.05
C8					0.95	0.94	0.96	0.73
C9					0.94	1.06	0.98	0.66
C10					0.95	0.96	0.96	0.71
C11					0.94	1.09	0.95	0.76
P1			0.91	0.89			0.92	0.81
P2			0.91	0.96			0.89	0.95
P3			0.85	1.50			0.83	1.32
P4			0.92	1.21			0.92	0.93
P5			0.93	0.82			0.91	0.89
P6			0.94	0.90			0.90	0.95
P7			0.89	1.17			0.84	1.33
P8			0.90	1.16			0.87	1.15
P9			0.92	0.83			0.90	0.88
P10			0.90	1.31			0.88	1.06
P11			0.97	0.81			0.97	0.70
P12			0.95	0.86			0.94	0.78
P13			0.94	1.08			0.92	0.87
P14			0.92	1.06			0.91	0.86
P15			0.97	0.64			0.98	0.57
P16			0.88	1.10			0.90	0.92
P17			0.94	0.77			0.91	0.75
P18			0.93	1.04			0.95	0.87
P19			0.90	0.98			0.91	0.83
P20			0.95	0.83			0.96	0.69
P21			0.85	1.18			0.84	1.16
P22			0.87	1.18			0.89	1.09
P23			0.87	1.12			0.90	0.97
P24			0.85	1.18			0.85	1.04

**Fig. 1:**
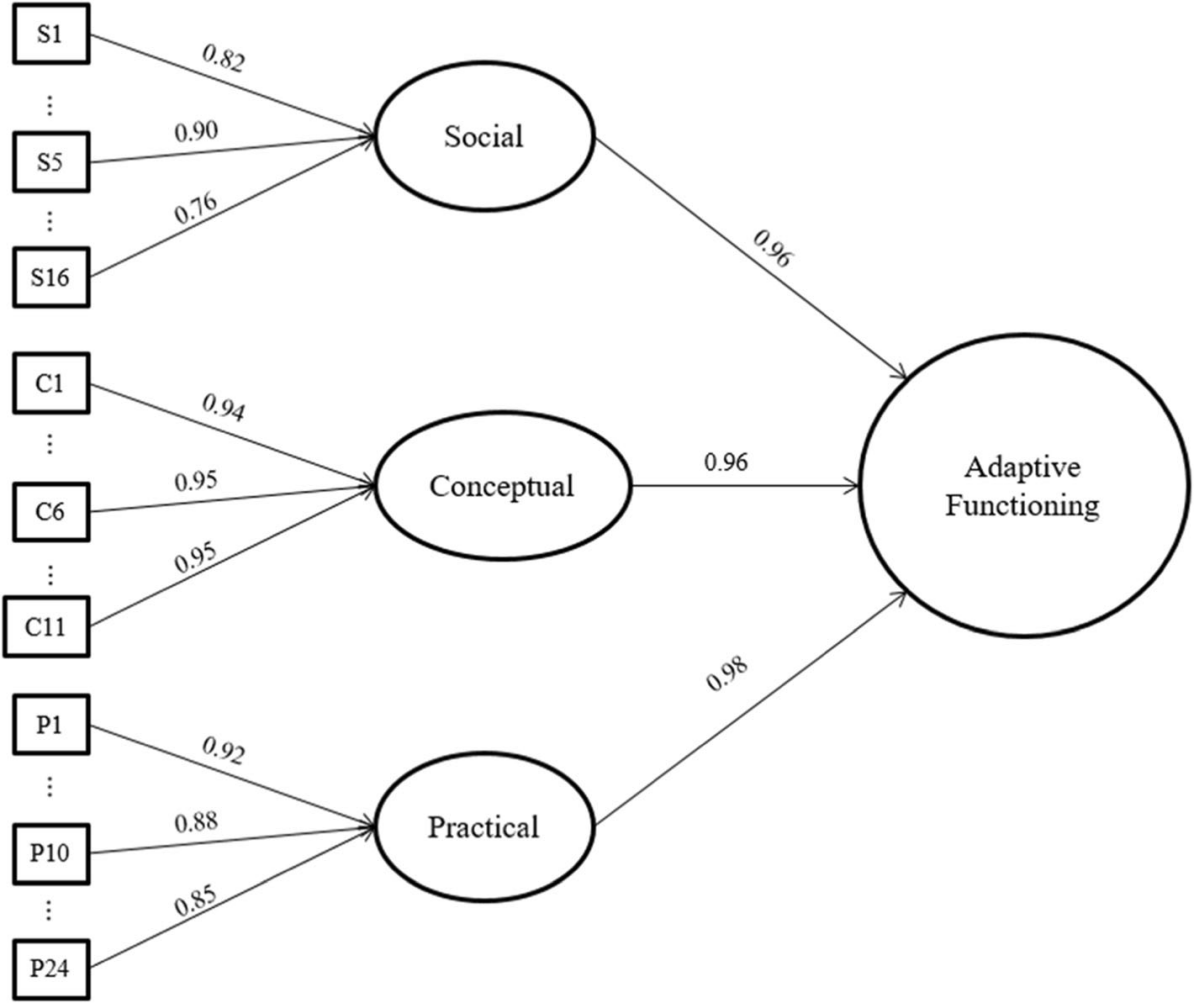
Confirmatory model considering an overall factor of adaptive functioning. Note: Some items were omitted to facilitate visualization of the model; the specified factor loadings of all the items in the model are presented in Table 3

According to Fig. 1, the social, conceptual, and practical domains presented high positive factor loadings in the overall factor of adaptive functioning. The latent variables loaded significantly in the second-order factor, with factor loadings that ranged from 0.96 to 0.98. Analysis of items’ adequacy to the measurement model using IRT analysis indicated that, after the changes, all the items presented acceptable parameters with infit values between 0.5 and 1.5 (Table 3).

**Fig. 2:**
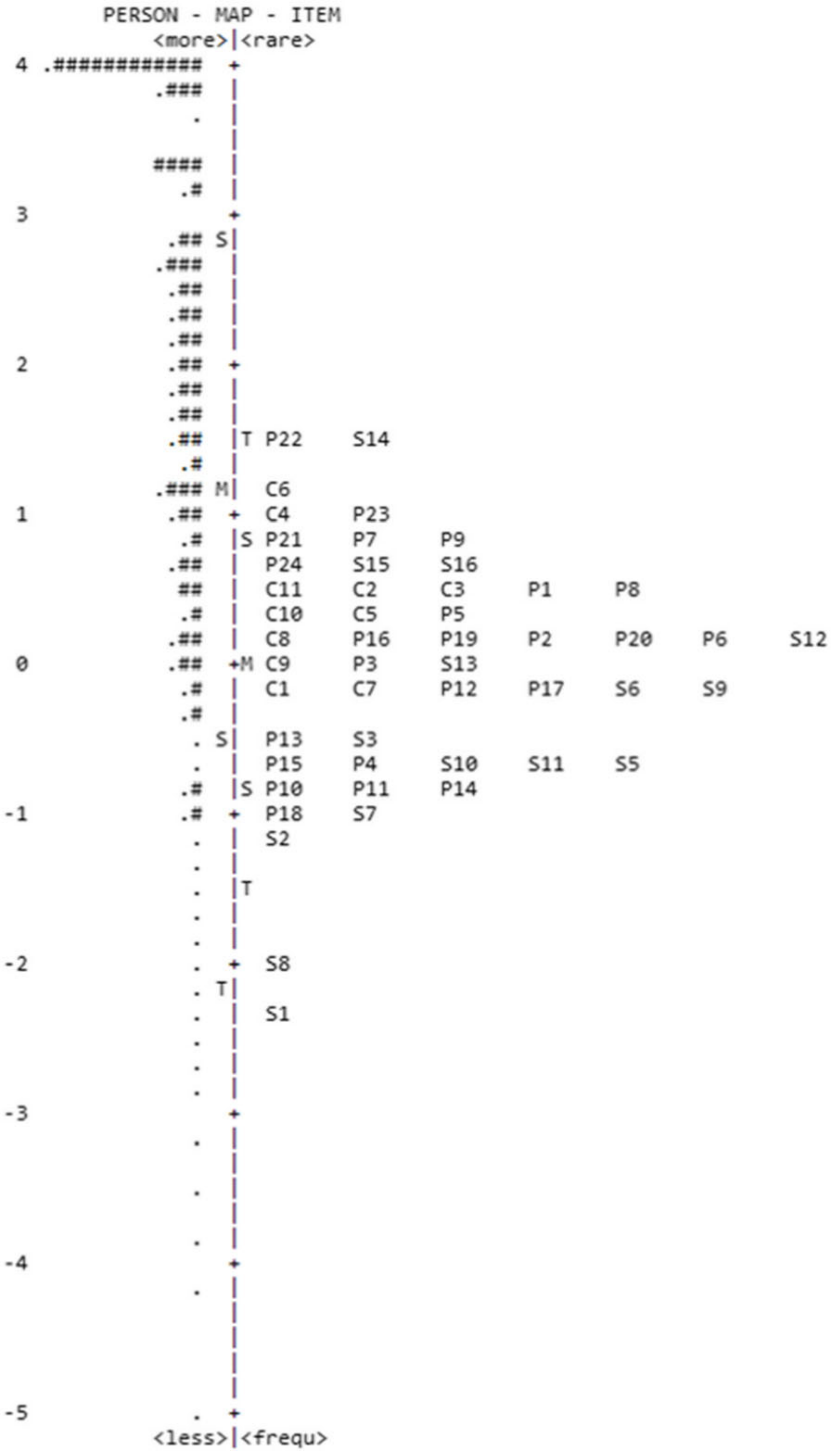
EFA-DI’s item-person map. M, mean; S, 1st standard deviation; T, 2nd standard deviation; number sign indicates group of seven people; full spot indicates group of six people

**Fig. 3:**
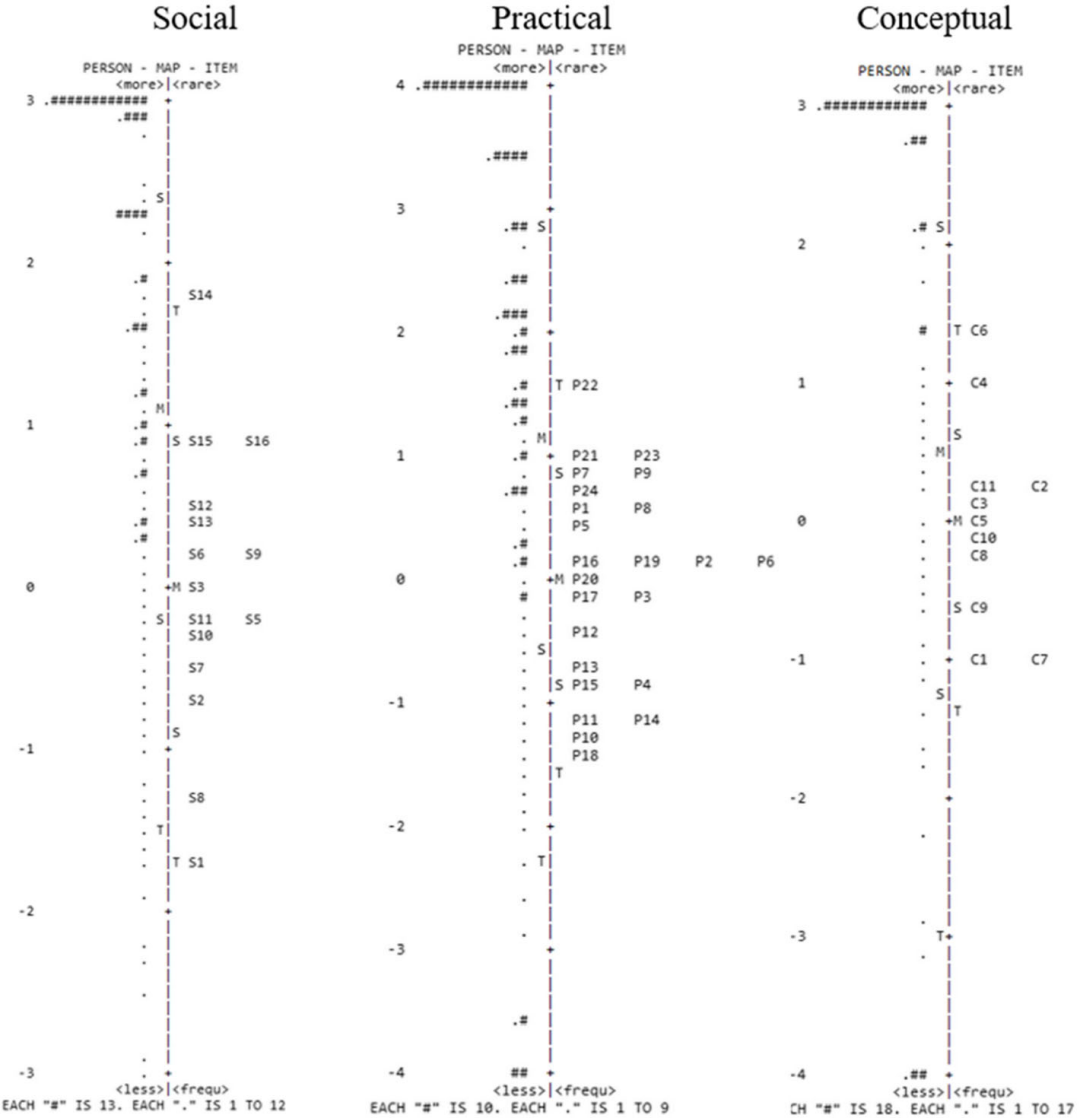
Item-person map of the EFA-DI’s social, practical, and conceptual domains. M, mean; S, 1st standard deviation; T, 2nd standard deviation; number sign indicates group of 15 people; full spots indicates group from 1 to 14 people

The adequacy of the items to the whole latent continuum was also assessed using an item-person map. The item-person map (Figs. 2 and 3) graphically presents the *logit* scores of children/adolescents and the items’ location along the latent trait of all the EFA-DI’s items. Regarding how precise the adaptive functioning estimates are, note that the scale has items that cover a large portion of participants’ skills. The participants’ skills in the EFA-DI, estimated using the Rasch model, ranged from 5.35 to 5.06 (*M* = 1.7; SD = 2.2), while difficulty in the items ranged from − 2.38 to 1.58 (*M* = − 0.84; SD = 0.49). In the social domain, the participants’ skills ranged from − 4.06 to 4 (*M* = 1.9; SD = 1.7), and the difficulty of items ranged from − 1.69 to 1.84 (*M* = − 0.40; SD = 1.82). In the practical domain, the participants’ skills ranged from − 4.79 to 4.63 (*M* = 1.7; SD = 2.4), and the difficulty of items ranged from − 1.38 to 1.56 (*M* = 0.66; SD = 0.09). The participants’ skills in the conceptual domain ranged from − 4.43 to 3.94 (*M* = 1.5; SD = 2.7), and difficulty in the items ranged from − 0.95 to 1.35 (*M* = − 0.33; SD = 0.87). A ceiling effect was found in the distribution of items and is presented in Figs. 2 and 3.

In addition to the analyses of validity related to internal consistency, the EFA-DI’s items were ordered according to the item-person map and level of difficulty presented. The authors opted to change the order of the scale based on this analysis.

Regarding the comparison of scores between the clinical and typical development groups, covariance analyses indicated significant differences given the clinical group reported, *F*(3.540) = 154.20, *p* < 0.001, regarding adaptive functioning levels. No differences were found in regard to the children/adolescents’ sex [*F*(1.540) = 2.09; *p* = 0.14], though a significant effect was found for the age covariate [(*F*(1.540) = 85.30; *p* < 0.001] (Table 4). Post hoc analyses indicated significant differences in all the groups, except for the moderate and severe/profound groups (Table 4).

**Table 4:** Bootstrapped adjusted means and post hoc analyses for EFA’s scores as a function of ID group

Groups	EFA-DI’s domains			
	Social *M (CI)*	Practical *M (CI)*	Conceptual *M (CI)*	Adaptive functioning *M (CI)*
Typical development	2.54 (2.42–2.68) ^abc^	2.65(2.49–2.81) ^abc^	2.77 (2.61–2.94)^abc^	2.60 (2.43–2.77)^abc^
Mild ID	0.43 (0.24–0.64) ^ade^	− 0.62 (− 1.41–0.26)^ade^	− 1.36 (− 1.86 to − 0.76) ^ade^	− 0.26 (− 0.63–0.22) ^ade^
Moderate ID	− 0.16 (− 0.58–0.25) ^bd^	− 1.45 (− 2.07 to − 0.84) ^bd^	− 2.41(− 2.98 to − 1.84) ^bd^	− 0.91 (− 1.29 to − 0.54) ^bd^
Severe/Profound ID	− 0.60 (− 1.21–0.14) ^ce^	− 2.35 (− 3.14 to − 1.45) ^ce^	− 3.14 (− 3.63 to − 2.55) ^ce^	− 1.52 (− 2.17 to − 0.93)^ce^

Means were adjusted for participants’ sex and age. The superscript letters identify the means for adaptive functioning that differed significantly (*p* < 0.05) from the type of diagnosis reported. Two equal letters in each column indicate that means are significantly different

*IC* 95% bootstrap confidence interval

## Discussion

This study’s objective was to assess the psychometric characteristics of the *Escala de Funcionamento Adaptativo para Deficiência Intelectual* (EFA-DI) [Adaptive Functioning Scale for Intellectual Disability], designed to assess the AF of 7- to 15-year-old children and adolescents. The procedures used here were intended to gather validity and reliability evidence following the standards for American Educational Research Association, American Psychological Association, & National Council on Measurement in Education [AERA, APA, & NCME] (2014). The analyses’ results indicate the EFA-DI’s initial psychometric quality to assess adaptive functioning, while the validity and reliability indexes confirm that the instrument can be used to evaluate AF.

The analyses to verify the scale’s dimension included confirmatory factor analysis of the three-domain theoretical model, considering an overall second-order factor. The results indicated a very high correlation between the scale’s domain and the second-order factor (0.96 to 0.98), suggesting a one-factor structure. Nonetheless, we opted to follow the theoretical model used in the development of the scale because it was confirmed in the analysis of unidimensionality and because it has practical relevance when establishing a comprehensive ID diagnosis. The EFA-DI was developed according to the theoretical conceptualization of adaptive functioning adopted by the DSM-5 (APA, 2013), considering the manual’s criteria for the intellectual disability diagnosis. The domains were chosen according to the same rationale and were divided into conceptual, social, and practical domains. For this reason, it was essential to verify whether the EFA-DI domains would also provide an overall score of adaptive functioning, specifying a second-order factor. The fit indexes confirmed that these domains are relevant for the investigation of adaptive functioning, in line with different guidelines that advocate considering the three-domain theoretical model (AAIDD, 2012; APA, 2013; Tassé et al., 2012).

According to the recommended parameters, only items S4—“*Uses gestures to communicate his/her needs and desires* (*e.g.*, *wags a finger to say no*; *points to something s/he wants*)” and C12—*Remains attentive in routine tasks* (*that is, does not lose focus during tasks*)—presented poor fit to the measurement model, according to the parameters required. A qualitative analysis showed that a high non-expected percentage of “No” answers were found—when the child/adolescent does not present a given behavior—compared to other items from the typical development sample. This high percentage may indicate that the participants failed to understand the items. In this case, the examples may have hindered understanding, as the item’s content is more comprehensive than the examples provided.

The removal of these items from the analysis of validity shows that even when we follow the procedures recommended in the literature to develop a scale (DeVellis, 2016; Pasquali, 2010) and perform a rigorous semantic analysis, the items may still be unfit when psychometric evaluated. Other studies highlight this same situation when addressing the development of instruments (Silva, 2017).

The inspection of the item-person map allowed the verification of the items’ discriminatory capacity in each domain. The results show that, in general, most of the sample was distributed within the test validity range (de Ayala, 2009). A large percentage of the participants, however, obtained high scores. This response pattern was expected as the non-clinical group, that is, individuals with typical development, composed most of the sample. The EFA-DI presents a few items that may be difficult for people with typical development. Note that the AF construct is not normally distributed in the population; i.e., high AF skills are not observed (Spreat, 2017). The AF distribution increasingly deviates from the normal curve as cognitive skills increase (Spreat, 2017), suggesting that AF is not normally distributed in populations with typical development. Hence, considering this expected ceiling effect, the EFA-DI represents this construct’s distribution in the population.

Spreat (2017) notes the practical implication of this finding in ID diagnosis, as the cutoff point of two standard deviations below the intelligence mean cannot probably be assumed for AF. Considering the scale’s objective, which is specific for the ID diagnosis, the results show that the items covered different skills’ levels from the clinical sample.

Analyses of criterion validity enabled exploring more deeply the EFA-DI’s practical potential as a psychological test in clinical use as a tool that allows the distinction of ID severity types. The severe ID and profound ID subsamples were gathered in the same group for these analyses, considering the similar level of assistance requirement of the individuals in these groups and the small sample size of individuals with profound ID in this study (*n* = 7). Both groups presented the lowest EFA-DI scores since (a) limited access to conceptual skills is expected, with limited comprehension ability, especially regarding symbolic processes; (b) limited expressive and comprehensive language; and (c) a need for support to perform daily tasks, possibly presenting maladaptive behaviors such as self-injurious behavior (AAIDD, 2012; APA, 2013).

The EFA-DI items presented an adequate capacity to discriminate between the non-clinical and clinical groups and between mild ID and moderate ID. The scale, however, did not differentiate between moderate ID and severe/profound ID. As psychologists find it challenging to distinguish mild and moderate ID in clinical practice (Silva & Silveira, 2019), EFA-DI is a promising instrument to support diagnostic accuracy. On the other hand, individuals with severe/profound ID face considerable difficulties performing daily tasks, being not testable in many cases, preventing the psychological assessment (APA, 2013).

The severe and profound levels of ID are often confused, mainly because there are many associated comorbidities. In this study, the participants with multiple disabilities may have worked as a confounder of more severe cases of ID and consequently hindered the scale’s discriminatory power.

Note that not having access to the participants’ medical records is one of this study’s limitations. The diagnoses were reported just by the respondents, which may have led to a prediction error concerning the level of severity of the ID. Future studies should control this variable in the data collection procedures, providing new evidence of criterion validity, especially relating to severe and profound levels of ID. Diagnosis is an important criterion to establish an instrument’s validity, as indicated in the specialized literature (APA, 2013; Pasquali, 2010). In this sense, the EFA-DI accumulates evidence of criterion validity concerning the diagnosis.

Another limitation is the broad age range in the sample, which may have led to the underestimation of some parameters. AF skills differ considerably according to age because they follow child development (AAIDD, 2012). We can assume that by restricting the age group, the items can better cover the skills continuum. Future studies with larger sample sizes will enable multi-group analyses, considering different age ranges and investigating new evidence of the EFA-DI validity.

Additionally, intellectual disability is a heterogeneous neurodevelopmental disorder associated with different genetic syndromes and frequently associated with other disorders, such as ASD and other medical conditions, such as physical disability or cerebral palsy (AAIDD, 2012; APA, 2013). These confounding factors may hinder an accurate assessment of ID severity. An individual who needs to use a wheelchair, for instance, will face limitations in daily tasks, from locomotion to personal hygiene care. Thus, when associated with an ID diagnosis, it is difficult to determine whether the level of assistance required is a consequence of a more severe ID or of the physical disability itself. An analysis of subgroups without multiple disabilities and without comorbidities, or few comorbidities, could control this variation and investigate these covariables’ impact.

Future studies addressing a more extensive and diverse sample, with specific clinical groups, and testing correlations with other instruments and reliability evidence will provide further evidence to support the use of the EFA-DI to assess adaptive functioning and support the diagnosis of ID. A study to develop standards to interpret the scale is already underway.

## Conclusion

Finally, we believe this study’s contributions exceed its limitations. The assessment of psychometric properties involved advanced statistical analyses such as IRT, while all the results provided validity evidence for the EFA-DI use to assess AF. Additionally, the scale’s different psychometric indicators were explored according to specialized literature (APA, 2013). Hence, the EFA-DI has the potential to, at least partially, fill in the gap of instruments to assess AF in the Brazilian context.

## Data Availability

The datasets used and/or analyzed during the current study are available from the corresponding author upon reasonable request.

## Abbreviations

AAIDD: American Association on Intellectual and Developmental Disabilities
AERA: American Educational Research Association
APA: American Psychological Association
ICD: International Classification of Diseases and Related Health Problems
ID: Intellectual disability
DSM: Diagnostic and Statistical Manual of Mental Disorders
EFA-DI: *Escala de Funcionamento Adaptativo para Deficiência Intelectual* [Adaptive Functioning Scale for Intellectual Disability]
AF: Adaptive functioning
GEAPAP: *Grupo de Estudo*, *Aplicação e Pesquisa em Avaliação Psicológica* [Psychological Assessment, Study, Application and Research Group]
NCME: National Council on Measurement in Education
CTT: Classical test theory
IRT: Item response theory
